# Validation of the Slovenian versions of Child and Youth Resilience Measure-12 and Brief Resilience Scale among youth

**DOI:** 10.3389/fpsyg.2025.1467174

**Published:** 2025-02-19

**Authors:** Urška Smrke, Ana Rehberger, Sara Močnik, Tanja Špes, Izidor Mlakar, Nejc Plohl

**Affiliations:** ^1^Laboratory for Digital Signal Processing, Faculty of Electrical Engineering and Computer Science, University of Maribor, Maribor, Slovenia; ^2^Unit for Paediatric and Adolescent Psychiatry, Division of Paediatrics, University Medical Centre Maribor, Maribor, Slovenia; ^3^Department of Psychology, Faculty of Arts, University of Maribor, Maribor, Slovenia; ^4^Department of Psychology, Faculty of Medicine, University of Maribor, Maribor, Slovenia

**Keywords:** resilience, validation, young adults, CYRM-12, BRS

## Abstract

**Introduction:**

Resilience is the ability to adapt positively in the face of adversity, trauma, or significant stress and is a vital component of maintaining mental health and well-being. It is particularly shaped in young adulthood by navigating unique stressors, such as changes in living arrangements, relationships, and education. However, much of the existing research focuses on children or older adults, leaving a gap in our knowledge regarding resilience in young adulthood. Moreover, the existing resilience scales are seldom validated outside of English-speaking contexts. With this paper, we turn attention to validating two resilience measures, Child and Youth Resilience Measure-12 (CYRM-12) and Brief Resilience Scale (BRS), in Slovenian language, using a sample of young adults.

**Method:**

We administered a survey among 330 young individuals (18–24 years) from Slovenia. Next to the central questionnaires, we also measured resilience with another scale, along with coping strategies, anxiety, depression, and quality of life.

**Results:**

For both resilience scales, one-factor structures fitted the data well and both scales demonstrated good internal consistency. CYRM-12 and BRS showed positive associations with another resilience scale and adaptive coping strategies, negative associations with anxiety, depression, and maladaptive coping strategies, and a unique contribution to predicting quality of life (with CYRM-12 demonstrating somewhat greater predictive value for quality of life than BRS), pointing to good convergent, discriminant, and incremental validity, respectively.

**Discussion:**

The results of our study suggest that CYRM-12 and BRS are both sufficiently reliable and valid for use among Slovenian young adults, with slightly stronger evidence supporting the validity of CYRM-12 compared to BRS.

## Introduction

Resilience, defined as the ability to adapt positively in the face of adversity, trauma, or significant stress (American Psychological Association, 2014; Southwick et al., 2014), is a vital component of maintaining mental health and well-being. Most people will encounter a traumatic experience at some point in their lives that can impact their mental health. Studying resilience is crucial because of its significant role in promoting psychological health, improving quality of life, and reducing the effects of stress and adverse experiences (Yıldırım and Green, 2024). These experiences can include tragedies, threats, or ongoing challenges such as bullying or difficult relationships. Exposure to such stress can lead to depression, anxiety, burnout, and even physical conditions like cardiovascular illnesses (Southwick et al., 2014). However, individuals differ significantly in how adversity affects them. Some experience severe long-term disruptions, while others face few or no long-term disruptions, and some even show improvement (Troy et al., 2023; Vásquez et al., 2024). Despite its importance, much remains to be understood about the mechanisms that promote resilience and how it can be effectively measured and strengthened.

Young adulthood is a particularly sensitive developmental stage marked by various new experiences that shape resilience, self-identity, and health habits, while also increasing stress (Zlotnick et al., 2022). This demographic often faces unique stressors such as changes in living arrangements, relationships, education, and employment. These significant transitions create instability and uncertainty, posing substantial mental health risks (Matud et al., 2020; Cassaretto et al., 2024). The 12-month prevalence of any psychiatric disorder exceeds 40% in individuals aged 19–29 (Arnett et al., 2014). From 2005 to 2017, the rates of major depressive episodes among those aged 18–25 increased from approximately 8% to over 13% (Twenge et al., 2019). Additionally, 31.4% of first-year students in the WHO World Mental Health International College Student project screened positive for at least one common DSM-IV anxiety, mood, or substance disorder (Auerbach et al., 2018). Understanding resilience in young adults can lead to long-term benefits, including improved life outcomes such as higher academic achievement, better career prospects, and overall life satisfaction.

There is a need for a better understanding of how resilience develops and operates in different contexts. Current research often focuses on children or older adults, leaving a gap in our knowledge about resilience during young adulthood. Moreover, while various scales measure resilience, their applicability and validity for different age groups and contexts require further investigation. In our research, we aimed to fill this gap by examining resilience in a sample of young adults. We have translated already established questionnaires that have so far never been used in our space and applied them on a Slovenian sample for the first time. We assessed the associations between resilience and other psychological constructs, such as coping strategies, anxiety, depression, and quality of life. By using these otherwise established resilience scales and investigating their factorial structure and validity in a new cultural context, we sought to contribute to the literature on resilience and its implications for young adult populations.

## Theoretical background

### Resilience

Resilience, shaped by both internal and external factors, has been explored through various theoretical frameworks. Potential determinants of resilience include genetic, epigenetic, developmental, demographic, cultural, economic, and social variables. It heavily relies on personal characteristics, external support systems, and coping strategies, and can be strengthened at multiple levels, including individual, family, community, and cultural contexts. Evidence suggests that resilience is linked to the ability to flexibly employ a range of coping strategies tailored to specific challenges and to use corrective feedback to refine those strategies (Southwick et al., 2014).

Coping strategies involve cognitive and behavioral efforts to manage demands that exceed personal resources (Lazarus and Folkman, 1984; Endler and Parker, 1990; Smith et al., 2016). Some strategies reduce stress and lead to positive outcomes, while others increase stress and result in negative outcomes (Parker and Endler, 1992; Endler and Parker, 1994; Smith et al., 2016). The effectiveness of a coping strategy can also depend on personal resilience (Lazarus and Folkman, 1984; Zeidner and Saklofske, 1996; Folkman and Moskowitz, 2000; Smith et al., 2016; Zengyan and Liu, 2024). Higher personal resilience is associated with greater use of task-oriented coping strategies, which in turn are linked to more adaptive outcomes. Conversely, higher resilience is linked to less reliance on nonconstructive emotion-oriented strategies, which are associated with poorer psychological outcomes (Smith et al., 2016).

### Existing scales of resilience for young adults

In a recent scoping review, Ballard et al. (2024) identified six commonly used self-report resilience scales for young adults - Connor-Davidson Resilience Scale (CD-RISC; Connor and Davidson, 2003), Resilience Scale (RS-25; Wagnild and Young, 1993), Child and Youth Resilience Measure (CYRM; Ungar and Liebenberg, 2011), Resiliency Scales for Children and Adolescents (RSCA; Prince-Embury, 2008), Resilience Scale for Adolescents (READ; Hjemdal et al., 2006) and the Brief Resilience Scale (BRS; Smith et al., 2008). Among these, CD-RISC and RS are most commonly used; however, they were initially designed for adult resilience research and later adapted for youth. In contrast, CYRM, RSCA, and READ were all developed specifically for children and adolescents (Ballard et al., 2024).

In our study, we focus on CYRM-12 and BRS due to their absence in the Slovenian context. The availability of additional widely utilized questionnaires would enable more straightforward comparisons of our findings with those of international studies, facilitating effective cross-cultural comparisons across diverse populations, aiding in understanding how resilience manifests itself in relation to other constructs in various contexts. For instance, the CYRM-12 has seldom been validated in studies conducted outside of English-speaking contexts (Mu and Hu, 2016). Furthermore, Ballard et al. (2024) reported that BRS has rarely been reviewed or applied in studies on adolescent resilience. Additionally, while CYRM-12 and BRS scales stem from different theoretical rationales for the construct of resilience (as described in the following sections), the relation between them (and to other resilience measures) has been rarely explored in empirical studies.

The CYRM was developed with the aim of capturing and understanding resilience across different cultures and contexts, based on a socio-ecological interpretation of resilience. During its development phase, a mixed-methods approach was employed, involving adolescents and adults from 11 countries (Ungar and Liebenberg, 2011). The original self-report scale (CYRM-28) measures three major categories of resilience: individual resources (personal skills, peer support, social skills), relationships with caregivers (physical and psychological caregiving) and contextual resources (spirituality, culture, education; Liebenberg et al., 2012). The shorter, 12-item measure can be used as a screening tool in clinical and non-clinical settings and is also suitable for inclusion in omnibus surveys. It was validated on a sample of adolescents exposed to higher risk as well as on a sample of school children aged 10–18 years. Data for certain analyses were collected from a sample of young people up to 22 years old. Liebenberg et al. (2013) report sufficient validity of the measure.

The BRS was developed to assess resilience as the ability to bounce back from stress. The reliability and validity of the scale was tested on four different samples, with two groups comprising students and two comprising adults afflicted with chronic conditions. The results suggested that BRS is a reliable and valid measure (Smith et al., 2008).

## Theoretically expected relations and hypotheses

### Psychometric structure of the central questionnaires

CYRM-12 was developed by Liebenberg et al. (2013), who proposed a model in which the 12 questionnaire items load onto a single latent factor. After allowing four pairs of items to covary, the one-factor structure exhibited satisfactory fit indices and reliability (for more information, see Liebenberg et al., 2013). Since then, the scale has been validated in several languages, for example Chinese (Mu and Hu, 2016) and Persian (Mohammadinia et al., 2024), with both studies supporting one-factor structure. In Arabic (Panter-Brick et al., 2021), the online version of CYRM-12 showed good validity and reliability. However, the factor structure was not tested. Supporting its multi-purpose aims, the questionnaire has also been validated in a disadvantaged community sample (Russell et al., 2021) and among Syrian refugee youth (Mohammadinia et al., 2024). Based on the one-factor structure of the original short-version questionnaire (Liebenberg et al., 2013) and further support for this structure in other languages, we set our first hypothesis below.

*H1: Slovenian version of CYRM-12 will exhibit a one-factor structure.*

The authors of BRS also proposed a one-factor structure of the questionnaire, which was tested and confirmed in their initial study (Smith et al., 2008) and other studies using English-speaking samples (e.g., McKay et al., 2019). So far, the measure has been translated and validated in many languages. Brazilian-Portuguese (da Silva-Sauer et al., 2020) and German (Chmitorz et al., 2018) validations showed a good fit of the unidimensional structure after accounting for another factor in the model only with negatively worded items. Similarly, Turkish (Haktanir et al., 2016), Korean (Choi et al., 2019), and French (Jacobs and Horsch, 2019) validations also showed a good fit of the unidimensional structure. On the other hand, validations on Spanish (Rodríguez-Rey et al., 2016), Greek (Kyriazos et al., 2018), Dutch (Soer et al., 2019), Chinese (Fung, 2020), Polish (Konaszewski et al., 2020), and Arabic (Baattaiah et al., 2023) samples tested a structure with two factors represented by positively and negatively worded items and showed a good fit. Still, since the initial study proposed a unidimensional structure, which has successfully been replicated in several studies, we set the second hypothesis.

*H2: Slovenian version of BRS will exhibit a one-factor structure.*

### Convergent validity: relations between resilience and related constructs

Smith et al. (2008) found a moderate positive correlation between the BRS scale and the CD-RISC scale. A moderate positive association was also found between CD-RISC and CYRM-12. Similarly, a moderate positive correlation between BRS and CYRM-12 was reported in Soliman. We expect the following associations between the resilience scales included in the study.

*H3: Resilience scales BRS and CYRM-12 will be positively related.*

*H4a: Resilience scale BRS will be positively related to another resilience scale, CD-RISC-10.*

*H4b: Resilience scale CYRM-12 will be positively related to another resilience scale, CD-RISC-10.*

As mentioned in the introduction, resilience is closely related to coping strategies skills (Southwick et al., 2014). Min et al. (2013) reported positive reappraisal and refocus on planning to be the two most important predictors of resilience among all tested coping strategies. Positive correlation with planning ranged from small to large, and positive correlation with positive reappraisal was in moderate range (Smith et al., 2008; Min et al., 2013; Lee et al., 2019). Following from this, we set hypotheses below predicting convergent validity of resilience scales.

*H5a: Resilience will be positively related with adaptive coping strategy*, i.e., *refocus on planning.*

*H5b: Resilience will be positively related with adaptive coping strategy*, i.e., *positive reappraisal.*

### Discriminant validity: relations between resilience and differing constructs

Following from defining resilience as the ability to adapt positively in the face of adversity or stress, previous research has found a negative association between resilience and mental health (Yıldırım et al., 2020). Resilience has been tested in the original article by Smith et al. (2008) on four different samples. Negative associations with both depression and anxiety ranged from moderate to large. Therefore, we set the two hypotheses below.

*H6: Resilience will be negatively related to depression.*

*H7: Resilience will be negatively related to anxiety.*

Similar to adaptive coping strategies, maladaptive coping strategies, such as self-blame and catastrophizing, also showed associations with resilience. Negative associations between resilience and self-blame ranged small to moderate (Lee et al., 2019; Min et al., 2013), while associations with catastrophizing were moderate negative (Min et al., 2013). Based on the associations found in the literature, we propose the following set of hypotheses linked to discriminant validity.

*H8a: Resilience will be negatively related with maladaptive coping strategy*, i.e., *self-blame.*

*H8b: Resilience will be negatively related with maladaptive coping strategy*, i.e., *catastrophizing.*

### Incremental validity: relation to quality of life as an outcome

When people assign positive meaning to negative experiences, which is indicative of high resilience, this act can have a positive effect on their perception of their position in life (Arslan and Yıldırım, 2021; Tempski et al., 2015). Previous studies have reported a significant positive association of resilience with all four subdimensions of quality of life; moderate association for physical and social, and large association for psychological and environmental quality of life (Keener et al., 2021), which is why we propose the same in our last hypothesis below.

*H9a: When controlling for other relevant* var*iables, resilience will be positively related to physical quality of life.*

*H9b: When controlling for other relevant variables, resilience will be positively related to psychological quality of life.*

*H9c: When controlling for other relevant variables, resilience will be positively related to social quality of life.*

*H9d: When controlling for other relevant variables, resilience will be positively related to environmental quality of life.*

## Aims of the study

Resilience plays an important role in maintaining and improving mental health, well-being, and quality of life. Especially young people face major changes and stressors that bring instability to their lives and constantly test their ability to bounce back from stress and drawbacks. Slovenian youth is no different in facing such challenges, thus we translated two scales, namely CYRM-12 and BRS, into the Slovenian language to be able to measure resilience in our youth. The aim of this study is to examine the internal and external validity of Slovenian versions of these two scales on a sample of Slovenian young adults. More specifically, we aimed to investigate the factorial structure and internal consistency of the two scales (i.e., CYRM-12 and BRS) and test the associations with other scales to explore various aspects of their validity. To explore convergent validity, we examine relations between three resilience measures (i.e., CYRM-12, BRS, and CD-RISC-10), and related constructs (i.e., two adaptive coping strategies, refocus on planning and positive reappraisal). To explore discriminant validity, we examine relations of resilience and differing constructs (i.e., depression, anxiety, and two maladaptive coping strategies, self-blame and catastrophizing). Finally, to explore incremental validity, we examine relations of resilience to quality of life as an outcome (i.e., physical, psychological, social, and environmental quality of life). With the results of this study, we will be able to determine whether CYRM-12 and BRS can be used in a Slovenian sample and provide reliable and valid information on resilience of Slovenian youth.

## Materials and methods

### Participants

A total of 389 participants started filling out the survey. Some had to be excluded from the final sample due to dropping out before finishing the survey (*n* = 21; 5.4%), not meeting age-related inclusion criteria (*n* = 11; 2.8%) or failing two or more attention checks (*n* = 27; 6.9%). The final sample, hence, consisted of 330 participants who attentively filled out all relevant questionnaires. Of those, 209 (63.3%) were female, 116 (35.2%) male, while 6 (1.7%) identified as ‘other’ or preferred not to answer the question about gender. Participants were, on average, 21.2 years old (SD = 1.85, min = 18, max = 24), and most of them had completed some form of secondary education (*n* = 229; 69.3%), followed by those with completed tertiary education (*n* = 75; 22.7%) and those with primary education (*n* = 26; 7.9%). The socioeconomic status of participants was, for the largest share of them, a lot lower than average (*n* = 145; 43.9%), followed by those with lower than average (*n* = 90; 27.3%), average (*n* = 76; 23.0%), higher (*n* = 15; 4.5%) and a lot higher (*n* = 4; 1.2%) than the average Slovenian net salary at the time of the study.

### Measures

#### Resilience

Resilience was assessed with two central questionnaires, specifically the Child and Youth Resilience Measure-12 (CYRM-12; Liebenberg et al., 2013), the Brief Resilience Scale (BRS; Smith et al., 2008).

#### Child and Youth Resilience Measure-12

CYRM-12 consists of 12 items (e.g., “I try to finish what I start”) that are answered on a 5-point scale (1 – “Does not describe me at all”, 5 – “Describes me a lot”). The scale exhibited good internal consistency in the validation study (α = 0.84).

#### Brief Resilience Scale

BRS consists of 6 items (e.g., “I tend to bounce back quickly after hard times”) that are answered on a 5-point scale (1 – “Strongly disagree”, 5 – “Strongly agree”). The scale exhibited good internal consistency in the validation study (α = 0.80–0.91).

Additionally, resilience was assessed with the Connor-Davidson Resilience Scale 10 (CD-RISC-10; Campbell-Stills and Stein, 2007; Kavčič et al., 2023). CD-RISC-10 consists of 10 items (e.g., “I am able to adapt when changes occur”) that are answered on a 5-point scale (0 – “Not true at all”, 4 – “True nearly all of the time”). The scale exhibited good internal consistency in the original validation study (α = 0.85) and a validation study of the Slovenian version (α = 0.83). Internal consistency coefficients obtained in the present study for all questionnaires are reported in the Results section.

### Coping strategies

Coping strategies were assessed via selected dimensions of Cognitive Emotional Regulation Questionnaire (CERQ; Garnefski et al., 2001), i.e., two dimensions assessing positive coping strategies, namely refocus on planning (2 items, e.g., “I think of what I can do best”) and positive reappraisal (2 items, e.g., “I think I can learn something from the situation”), and two assessing negative coping strategies, namely self-blame (2 items, e.g., “I think about the mistakes I have made in this matter”) and catastrophizing (2 items, e.g., “I continually think how horrible the situation has been”). Items are answered on a 5-point scale (1 – “(Almost) never”, 5 – “(Almost) always”). The scale exhibited satisfactory internal consistency in the original validation study for all dimensions used in this study (α = 0.72–0.81).

### Anxiety

Anxiety was assessed with the Generalized anxiety disorder questionnaire (GAD7; Spitzer et al., 2006; Jelenko Roth and Dernovšek, 2011). It consists of 7 items asking about the frequency of generalized anxiety disorder symptoms that participants were bothered by in the last 2 weeks (e.g., “Worrying too much about different things”). Items are answered on a 4-point scale (0 – “Not at all”, 3 – “Nearly every day”). The scale exhibited excellent internal consistency in the original validation study (α = 0.92).

### Depression

Depression was measured with the Patient Health Questionnaire (PHQ-9; Kroenke et al., 2001; Cesar et al., 2021) that consists of 9 items asking about the frequency of symptoms of depression that participants were bothered by in the last 2 weeks (e.g., “Feeling tired or having little energy”). Items are answered on a 4-point scale (0 – “Not at all”, 3 – “Nearly every day”). The scale exhibited good internal consistency in the original validation study (α = 0.86–0.89).

### Quality of life

Quality of life (QoL) was measured with the World Health Organization Quality-of-Life Scale (WHOQOL-BREF; WHOQOL Group, 1998). It consists of 26 items. 24 items form domain scores that reflect individuals’ perceptions of their psychological QoL (7 items, e.g., “How much do you enjoy life?”), physical QoL (6 items, e.g., “Do you have enough energy for everyday life?”), social relationships (3 items, e.g., “How satisfied are you with your personal relationships?”), and environment (8 items, e.g., “How healthy is your physical environment?”). The remaining two items refer to the overall perception of quality of life and health, respectively, and were omitted from the analyses of this study. Items are answered on a 5-point scale with labels varying between different subsets of items (e.g., 1 – “Very poor”, 5 – “Very good”; 1 – “Not at all”, 5 – “An extreme amount”). The scale exhibited acceptable to good internal consistency in the validation study (physical: α = 0.80–0.84; psychological: α = 0.75–0.77; social relationships: α = 0.66–0.69; environment α = 0.80).

### Additional questions

In addition to the questionnaires described above, participants were asked to provide information on their socio-demographic characteristics and respond to three attention check items spread throughout the survey, all in the form of directed questions (e.g., “This is a control question. Mark “Strongly disagree” and continue”; Maniaci and Rogge, 2014).

### Procedure

The authors translated all questionnaires that were not already available in Slovenian (i.e., CYRM-12, BRS, and WHOQOL-BREF) using the translation-back translation method. Specifically, two researchers independently translated the items into Slovenian, and these translations were merged by a third researcher. The merged Slovenian items were translated back to the original language by a fourth researcher who was not familiar with the original items. The original and back-translated items were compared, with all discrepancies resolved by discussion. The translators had a psychology or psychiatry background with additional expertise in the social sciences methodology and had (close-to) native proficiency in both languages.

The study was conducted in Slovenia. The participants were recruited via the Valicon online panel^1^ that is focused on marketing and other types of research and data collection, primarily in Slovenia. The data was collected in March and April 2024. Participants had to be between 18 and 24 years old to be eligible for participation (*M* = 21.2, SD = 1.85). First, they were informed about the aims and characteristics of the study and on the voluntary and anonymous nature of their participation. After consenting to participate, they proceeded to an online survey on the 1 ka platform.^2^ The survey took 16.5 min to complete on average. Participants received small monetary compensation for their participation as agreed between them and the online panel. Ethical approval was not sought for this study, as per institutional and national guidelines.

### Statistical analyses

We first cleaned the database by excluding participants who did not meet our inclusion criteria, dropped out during the study, or failed more than one attention check. As the remaining participants had no missing values, no data needed to be imputed. We also checked for common method bias by calculating Harman’s single factor score that loads all items used in the study onto a single factor. The results showed that the single factor explains 26.90% of total variance, which is below the recommended threshold of 50.00% (Harman, 1960). As such, there is no evidence of common method bias in our study.

In the next step, we calculated the relevant descriptive statistics, tested the assumptions, and conducted confirmatory factor analyses (CFA) in the Mplus 8.0 software. Maximum Likelihood (ML) was used as the default estimator. However, since some items of the CYRM-12 exhibited larger deviations from the normal distribution, ML was replaced with the unweighted least squares (ULS) estimator in confirmatory factor analyses of this questionnaire (Li, 2016). The model fit was assessed by examining the Comparative Fit Index (recommended CFI ≥ 0.90), Tucker-Lewis Index (recommended TLI ≥ 0.90), Root Mean Square Error of Approximation (recommended RMSEA ≤0.080), and Standardized Root Mean Residual (recommended SRMR ≤0.080; Kline, 2005). We additionally investigated the factor loadings and standard errors (SE). Moreover, we investigated the modification indices (MI) to identify potential areas of misfit.

The remaining analyses were conducted in IBM SPSS Statistics 28. We first assessed the internal consistency of the central measures and other included questionnaires with α coefficients. Next, we calculated the factor scores, descriptive statistics, and bivariate associations between the constructs (using Pearson’s r). Lastly, we conducted multiple regression analyses to investigate the predictive value of the central questionnaires in predicting different quality of life aspects (controlling for other predictors of these outcomes).

## Results

### Factorial structure and internal consistency of the central measures

#### Child and Youth Resilience Measure-12

Prior to conducting confirmatory factor analyses, we performed the Kaiser-Meyer-Olkin test and Bartlett’s test of sphericity, which showed sampling adequacy (overall KMO = 0.89; KMO of individual items >0.81) and sufficiently large correlations among items (χ2(66) = 1387.26, *p* < 0.001). Since skewness and kurtosis (Table 1) indicated larger deviations from the normal distribution in the case of some items (i.e., items 2 and 5), we applied the unweighted least squares (ULS) estimator when investigating the factorial structure.

**Table 1:** Descriptive statistics of the CYRM-12 items and standardized factor loadings.

Item	Min	Max	*M*	*SD*	*S*	*K*	Factor loading (*SE*)
1. I have people I look up to.	1.00	5.00	3.14	1.08	−0.10	−0.70	0.44 (0.03)
2. Getting an education is important to me.	1.00	5.00	4.09	1.02	−1.14	0.78	0.42 (0.03)
3. My parent(s)/caregiver(s) know a lot about me.	1.00	5.00	3.46	1.16	−0.49	−0.54	0.58 (0.03)
4. I try to finish what I start.	1.00	5.00	3.98	0.93	−0.81	0.52	0.45 (0.03)
5. I am able to solve problems without harming myself or others (for example by using drugs and/or being violent).	1.00	5.00	4.47	0.85	−1.85	3.55	0.37 (0.03)
6. I know where to go in my community to get help.	1.00	5.00	3.56	1.13	−0.45	−0.51	0.80 (0.03)
7. I feel I belong at my school.	1.00	5.00	3.21	1.24	−0.23	−0.91	0.86 (0.03)
8. My family stands by me during difficult times.	1.00	5.00	4.00	1.13	−0.95	−0.09	0.69 (0.03)
9. My friends stand by me during difficult times.	1.00	5.00	3.87	1.15	−0.80	−0.23	0.80 (0.03)
10. I am treated fairly in my community.	1.00	5.00	3.82	0.95	−0.84	0.57	0.71 (0.03)
11. I have opportunities to develop skills that will be useful later in life (like job skills and skills to care for others).	1.00	5.00	3.93	1.01	−0.82	0.36	0.70 (0.03)
12. I enjoy my community’s traditions.	1.00	5.00	3.61	1.04	−0.58	−0.04	0.69 (0.03)

All standardized factor loadings were statistically significant (*p* < 0.001).

The proposed one-factor model fitted the observed data well: CFI = 0.981, TLI = 0.976, RMSEA = 0.065 (90% CI = [0.051; 0.080]), SRMR = 0.064. However, further analyses revealed a very high modification index (MI = 51.53) pertaining to the residual covariance between items 3 and 8, both related to individuals’ perceptions of their families. The modified model that allowed residuals of items 3 and 8 to be correlated improved the fit: CFI = 0.994, TLI = 0.992, RMSEA = 0.038 (90% CI = [0.017; 0.055]), SRMR = 0.054. As the remaining modification indices were much lower (MI < 8.00), we did not make any further modifications. The standardized factor loadings were statistically significant and ranged from 0.38 to 0.86 (Table 1). Moreover, the internal consistency of the scale was great (α = 0.87).

#### Brief Resilience Scale

First, we performed the Kaiser-Meyer-Olkin test and Bartlett’s test of sphericity, which showed sampling adequacy (overall KMO = 0.84; KMO of individual items >0.81) and sufficiently large correlations among items (χ2(15) = 537.66, *p* < 0.001). As skewness and kurtosis (Table 2) did not indicate any considerable deviations from the normal distribution, we used the maximum likelihood (ML) estimator in further analyses.

**Table 2:** Descriptive statistics of the BRS items and standardized factor loadings.

Item	Min	Max	*M*	*SD*	*S*	*K*	Factor loading (*SE*)
1. I tend to bounce back quickly after hard times.	1.00	5.00	3.36	1.03	−0.38	−0.49	0.66 (0.06)
2. I have a hard time making it through stressful events.^a^	1.00	5.00	2.96	0.99	−0.08	−0.99	0.68 (0.05)
3. It does not take me long to recover from a stressful event.	1.00	5.00	3.12	1.05	0.03	−0.94	0.74 (0.06)
4. It is hard for me to snap back when something bad happens.^a^	1.00	5.00	3.12	0.93	−0.23	−1.00	0.69 (0.05)
5. I usually come through difficult times with little trouble.	1.00	5.00	2.96	0.88	0.17	−0.63	0.38 (0.05)
6. I tend to take a long time to get over setbacks in my life.^a^	1.00	5.00	2.97	1.03	−0.13	−0.86	0.61 (0.06)

All standardized factor loadings were statistically significant (*p* < 0.001). ^a^Items were reversed.

The proposed one-factor model fitted the observed data well: CFI = 0.965, TLI = 0.942, RMSEA = 0.079 (90% CI = [0.046; 0.114]), SRMR = 0.037. Since we did not observe any exceptionally high modification indices, we did not adjust the initial model. The standardized factor loadings were statistically significant and ranged from 0.38 to 0.74 (Table 2). Additionally, the internal consistency of the scale was good (α = 0.80).

### Convergent, discriminant, and incremental validity of the central measures

We tested the convergent, discriminant, and incremental validity by calculating the bivariate Pearson’s correlations between the CYRM-12 and BRS scores and other relevant constructs and performing hierarchical regression analyses.

#### Convergent validity

The results showed that CYRM-12 and BRS were moderately positively associated (*r* = 0.32, *p* < 0.001), and both CYRM-12 (*r* = 0.60, *p* < 0.001) and BRS (*r* = 0.60, *p* < 0.001) were strongly positively associated with another measure of resilience, CD-RISC-10. Moreover, we found weak to intermediate positive associations between CYRM-12 and the two adaptive coping strategies (refocus on planning: *r* = 0.27, *p* < 0.001; positive reappraisal: *r* = 0.49, *p* < 0.001). While somewhat weaker in strength, the associations between BRS and the two adaptive coping strategies were also positive and ranged from weak to intermediate (refocus on planning: *r* = 0.13, *p* = 0.021; positive reappraisal: *r* = 0.32, *p* < 0.001). All bivariate associations were statistically significant.

#### Discriminant validity

Regarding discriminant validity, the results showed weak to intermediate negative associations between CYRM-12 and the two maladaptive coping strategies (self-blame: *r* = −0.15, *p* = 0.005; catastrophizing: *r* = −0.30, *p* < 0.001). Similarly, the associations between BRS and the two maladaptive coping strategies were negative and weak to intermediate in strength (self-blame: *r* = −0.20, *p* < 0.001; catastrophizing: *r* = −0.44, *p* < 0.001). Furthermore, CYRM-12 (*r* = −0.47, *p* < 0.001) and BRS (*r* = −0.48, *p* < 0.001) were both intermediately and negatively associated with depression. While the association between the two central questionnaires and anxiety was also negative, it ranged from intermediate in the case of CYRM-12 (*r* = −0.37, *p* < 0.001) to strong in the case of BRS (*r* = −0.52, *p* < 0.001). All reported bivariate associations were statistically significant.

#### Associations with outcomes and incremental validity

The results showed that CYRM-12 was strongly positively associated with all four quality of life aspects (physical: *r* = 0.51, *p* < 0.001; psychological: *r* = 0.65, *p* < 0.001; social: *r* = 0.58, *p* < 0.001; environmental: *r* = 0.54, *p* < 0.001). On the other hand, the associations between BRS and different quality of life dimensions ranged from weak to strong (physical: *r* = 0.42, *p* < 0.001; psychological: *r* = 0.54, *p* < 0.001; social: *r* = 0.29, *p* < 0.001; environmental: *r* = 0.41, *p* < 0.001). All bivariate associations were again statistically significant (Table 3).

**Table 3:** Descriptive statistics and bivariate associations between constructs.

	*M* (*SD*)	1	2	3	4	5	6	7	8	9	10	11	12	13
1. Resilience (CYRM-12)	3.76 (0.68)	(0.87)												
2. Resilience (BRS)	3.08 (0.70)	0.32^***^	(0.80)											
3. Resilience (CD-RISC-10)	3.49 (0.70)	0.60^***^	0.60^***^	(0.88)										
4. Refocus on planning	3.57 (0.75)	0.27^***^	0.13^*^	0.33^***^	(0.61)									
5. Positive reappraisal	3.77 (0.89)	0.49^***^	0.32^***^	0.62^***^	0.39^***^	(0.81)								
6. Self-blame	3.27 (0.88)	−0.15^**^	−0.20^***^	−0.17^**^	0.17^**^	−0.03	(0.69)							
7. Catastrophizing	2.78 (0.95)	−0.30^***^	−0.44^***^	−0.40^***^	0.00	−0.24^***^	0.33^***^	(0.78)						
8. Depression	1.93 (0.68)	−0.47^***^	−0.48^***^	−0.53^***^	−0.12^*^	−0.30^***^	0.29^***^	0.39^***^	(0.90)					
9. Anxiety	2.04 (0.77)	−0.37^***^	−0.52^***^	−0.50^***^	−0.03	−0.24^***^	0.25^***^	0.45^***^	0.77^***^	(0.91)				
10. QOL: Physical	3.62 (0.61)	0.51^***^	0.42^***^	0.50^***^	0.09	0.34^***^	−0.23^***^	−0.37^***^	−0.64^***^	−0.47^***^	(0.76)			
11. QOL: Psychological	3.31 (0.76)	0.65^***^	0.54^***^	0.67^***^	0.20^***^	0.49^***^	−0.29^***^	−0.41^***^	−0.72^***^	−0.60^***^	0.68^***^	(0.85)		
12. QOL: Social	3.38 (0.84)	0.58^***^	0.29^***^	0.44^***^	0.13^*^	0.34^***^	−0.18^**^	−0.27^***^	−0.40^***^	−0.32^***^	0.44^***^	0.59^***^	(0.65)	
13. QOL: Environmental	3.62 (0.60)	0.54^***^	0.41^***^	0.45^***^	0.16^**^	0.26^***^	−0.17^**^	−0.30^***^	−0.42^***^	−0.38^***^	0.56^***^	0.57^***^	0.43^***^	(0.80)

*p < 0.050, **p < 0.010, ***p < 0.001.

The incremental validity of the two central questionnaires was investigated using hierarchical multiple regression with three steps. In Step 1, we added demographic variables (i.e., gender, age, education, and socio-economic status). In Step 2, we added the four coping strategies (i.e., refocus on planning. Positive reappraisal, self-blame, and catastrophizing). Lastly, in Step 3, we added one of the two central questionnaires (i.e., either CYRM-12 or BRS). Different dimensions of quality of life were treated as outcomes. The results of these analyses are displayed in Table 4 (CYRM-12) and Table 5 (BRS), while the results of exploratory analyses, in which we additionally controlled for CD-RISC-10, are displayed in Supplementary Tables S1, S2.

**Table 4:** Incremental validity of CYRM-12 in predicting quality of life.

	QOL: Physical			QOL: Psychological			QOL: Social			QOL: Environmental		
	S1	S2	S3	S1	S2	S3	S1	S2	S3	S1	S2	S3
Gender	−0.25^***^	−0.18^***^	−0.21^***^	−0.15^**^	−0.07	−0.11^**^	0.07	0.13^*^	0.09	−0.11^*^	−0.05	−0.10^*^
Age	−0.04	−0.05	−0.01	0.00	−0.01	0.04	0.03	0.02	0.07	−0.08	−0.08	−0.03
Education	0.08	0.07	0.02	0.06	0.05	−0.01	0.08	0.07	0.00	0.05	0.04	−0.03
Socioeconomic status	0.15^**^	0.10^*^	0.09^*^	0.16^**^	0.09^*^	0.08	0.07	0.02	0.01	0.24^***^	0.21^***^	0.20^***^
Refocus on planning		0.01	−0.04		0.08	0.02		0.04	−0.03		0.12^*^	0.05
Positive reappraisal		0.27^***^	0.12^*^		0.38^***^	0.20^***^		0.27^***^	0.08		0.14^*^	−0.05
Self-blame		−0.10	−0.06		−0.18^***^	−0.13^**^		−0.12^*^	−0.07		−0.07	−0.02
Catastrophizing		−0.23^***^	−0.15^**^		−0.25^***^	−0.16^***^		−0.20^***^	−0.10		−0.21^***^	−0.11^*^
Resilience (CYRM-12)			0.40^***^			0.47^***^			0.51^***^			0.50^***^
*R* ^2^	0.09	0.27	0.38	0.05	0.38	0.53	0.02	0.19	0.36	0.08	0.20	0.37
*F* ^a^	8.24^***^	14.51^***^	21.02^***^	4.47^**^	24.46^***^	39.75^***^	1.59	9.20^***^	20.07^***^	6.94^***^	9.69^***^	20.41^***^
*ΔR* ^2^	0.09	0.18	0.11	0.05	0.33	0.15	0.02	0.17	0.18	0.08	0.12	0.17
*ΔF* ^b^	8.24^***^	18.93^***^	53.73^***^	4.47^**^	42.18^***^	100.49^***^	1.59	16.50^***^	86.96^***^	6.94^***^	11.52^***^	85.47^***^

*N* = 325 as we only included those who identified as male (0) or female (1) in these analyses. S1 = Step 1, S2 = Step 2, S3 = Step 3. ^a^Degrees of freedom were 4, 320 in Step 1; 8, 316 in Step 2; and 9, 315 in Step 3. ^b^Degrees of freedom were 4, 320 in Step 1; 4, 316 in Step 2; and 1, 315 in Step 3. *p < 0.050, **p < 0.010, ***p < 0.001.

**Table 5:** Incremental validity of BRS in predicting quality of life.

	QOL: Physical			QOL: Psychological			QOL: Social			QOL: Environmental		
	S1	S2	S3	S1	S2	S3	S1	S2	S3	S1	S2	S3
Gender	−0.25^***^	−0.18^***^	−0.16^**^	−0.15^**^	−0.07	−0.03	0.07	0.13^*^	0.15^**^	−0.11^*^	−0.05	−0.02
Age	−0.04	−0.05	−0.03	0.00	−0.01	0.01	0.03	0.02	0.03	−0.08	−0.08	−0.06
Education	0.08	0.07	0.07	0.06	0.05	0.05	0.08	0.07	0.07	0.05	0.04	0.04
Socioeconomic status	0.15^**^	0.10^*^	0.08	0.16^**^	0.09^*^	0.05	0.07	0.02	0.00	0.24^***^	0.21^***^	0.18^***^
Refocus on planning		0.01	0.00		0.08	0.06		0.04	0.03		0.12^*^	0.11
Positive reappraisal		0.27^***^	0.23^***^		0.38^***^	0.32^***^		0.27^***^	0.24^***^		0.14^*^	0.09
Self-blame		−0.10	−0.09		−0.18^***^	−0.17^***^		−0.12^*^	−0.11^*^		−0.07	−0.06
Catastrophizing		−0.23^***^	−0.16^**^		−0.25^***^	−0.14^**^		−0.20^***^	−0.14^*^		−0.21^***^	−0.12^*^
Resilience (BRS)			0.21^***^			0.32^***^			0.16^**^			0.26^***^
*R* ^2^	0.09	0.27	0.30	0.05	0.38	0.45	0.02	0.19	0.21	0.08	0.20	0.24
*F* ^a^	8.24^***^	14.51^***^	14.91^***^	4.47^**^	24.46^***^	29.15^***^	1.59	9.20^***^	9.13^***^	6.94^***^	9.69^***^	11.30^***^
*ΔR* ^2^	0.09	0.18	0.03	0.05	0.33	0.07	0.02	0.17	0.02	0.08	0.12	0.05
*ΔF* ^b^	8.24^***^	18.93^***^	13.48^***^	4.47^**^	42.18^***^	41.55^***^	1.59	16.50^***^	7.14^**^	6.94^***^	11.52^***^	19.61^***^

*N* = 325 as we only included those who identified as male (0) or female (1) in these analyses. S1 = Step 1, S2 = Step 2, S3 = Step 3. ^a^Degrees of freedom were 4, 320 in Step 1; 8, 316 in Step 2; and 9, 315 in Step 3. ^b^Degrees of freedom were 4, 320 in Step 1; 4, 316 in Step 2; and 1, 315 in Step 3. *p < 0.050, **p < 0.010, ***p < 0.001.

The results suggest that CYRM-12 explained significant variance in all four quality of life aspects over and above demographic variables and the included coping strategies. Even after controlling for other variables, CYRM-12 was a moderate to strong predictor of physical (*β* = 0.40, *p* < 0.001), psychological (*β* = 0.47, *p* < 0.001), social (*β* = 0.51, *p* < 0.001), and environmental (*β* = 0.50, *p* < 0.001) quality of life. It explained an additional 10.7–17.5% of the variance. Similarly, BRS explained significant variance over and above demographic variables and the included coping strategies in physical (*β* = 0.21, *p* < 0.001), psychological (*β* = 0.32, *p* < 0.001), social (*β* = 0.16, *p* = 0.008), and environmental (*β* = 0.26, *p* < 0.001) quality of life. The amount of variance additionally explained by BRS was lower and ranged from 1.8 to 7.2%. It is worth noting that CYRM-12 also exhibited incremental validity in predicting the four outcomes when we added the fourth step (i.e., controlling for another measure of resilience), whereas BRS significantly predicted only psychological and environmental quality of life in these analyses (see Supplementary Tables S1, S2).

## Discussion

The present study aimed to investigate the factorial structure and internal consistency of the Slovenian versions of two resilience measures, CYRM-12 and BRS, and explore their convergent, discriminant, and incremental validity. To do so, we conducted an online study among Slovenian youth that yielded findings supporting the hypothesized factorial structure of both questionnaires, high internal reliability, as well as sufficient convergent, discriminant, and incremental validity. Table 6 presents the summary of the hypotheses of this study and whether they have received support following the analyses.

**Table 6:** Summary of hypotheses and their support by the results of the present study.

Hypothesis	Supported by the results of the present study
H1: Slovenian version of CYRM-12 will exhibit a one-factor structure.	Yes
H2: Slovenian version of BRS will exhibit a one-factor structure.	Yes
H3: Resilience scales BRS and CYRM-12 will be positively related.	Yes
H4a: Resilience scale BRS will be positively related to another resilience scale, CD-RISC-10.	Yes
H4b: Resilience scale CYRM-12 will be positively related to another resilience scale, CD-RISC-10.	Yes
H5a: Resilience will be positively related with adaptive coping strategy, i.e., refocus on planning.	Yes
H5b: Resilience will be positively related with adaptive coping strategy, i.e., positive reappraisal.	Yes
H6: Resilience will be negatively related to depression.	Yes
H7: Resilience will be negatively related to anxiety.	Yes
H8a: Resilience will be negatively related with maladaptive coping strategy, i.e., self-blame.	Yes
H8b: Resilience will be negatively related with maladaptive coping strategy, i.e., catastrophizing.	Yes
H9a: When controlling for other relevant variables, resilience will be positively related to physical quality of life.	Yes
H9b: When controlling for other relevant variables, resilience will be positively related to psychological quality of life.	Yes
H9c: When controlling for other relevant variables, resilience will be positively related to social quality of life.	Yes
H9d: When controlling for other relevant variables, resilience will be positively related to environmental quality of life.	Yes

First, we evaluated whether data obtained on a young Slovenian sample exhibited the same latent structure as in previous studies testing other language versions and focusing on other population segments. Our results supported the hypothesized (H1) one-factor structure of the CYRM-12. In particular, the proposed factorial structure resulted in satisfactory fit indices and factor loadings of all items. Despite that, we made one post-hoc adjustment to the model based on modification indices. Specifically, we allowed residuals of items 3 and 8, both related to individuals’ perception of their family, to covary, which further improved the fit. The support for the one-factor structure is aligned with previous research, including the original validation study (Liebenberg et al., 2013) and validations in other languages, such as Chinese (Mu and Hu, 2016) and Persian (Mohammadinia et al., 2024). Interestingly, similarly to our study, the original authors also allowed items “My parent(s)/caregiver(s) know a lot about me” and “My family stands by me during difficult times” to covary, but also made two additional adjustments based on modification indices that were not needed in our sample. The Slovenian version of the CYRM-12 also exhibited great internal reliability (0.87), comparable to the internal reliability observed in the original validation study (0.84; Liebenberg et al., 2013).

Furthermore, although the literature is not entirely consistent, with some studies proposing a multidimensional nature of BRS (e.g., Kyriazos et al., 2018; Rodríguez-Rey et al., 2016; Soer et al., 2019), our results supported the hypothesized (H2) unidimensional structure of this scale as well. Like the original validation study (Smith et al., 2008) and most validations in other languages, such as German (Chmitorz et al., 2018), French (Jacobs and Horsch, 2019), and Brazilian-Portuguese (da Silva-Sauer et al., 2020), the proposed one-factor model fitted our data well, without any need for further modifications. The Slovenian version of the BRS was also found to be internally consistent (0.80), with the α value being similar to the original validation study, where α ranged from 0.80 to 0.91 (Smith et al., 2008). Altogether, these results suggest that Slovene versions of both measures are sufficiently reliable and can confidently be treated as unidimensional.

After exploring factorial structures and internal consistencies of the central measures, we focused on the nomological network of resilience assessed with BRS and CYRM-12. Within the convergent validity domain, results supported our hypotheses. More specifically, both central measures, i.e., BRS and CYRM-12, were positively related (H3), as were they to the CD-RISC-10 resilience scale (H4a and H4b), which is in line with previous studies observing positive associations between these three measures (Smith et al., 2008; Soliman, 2017; Robinson et al., 2022). Both central measures were also positively associated with two adaptive coping strategies (i.e., refocus on planning and positive reappraisal), supporting our hypotheses (H5a and H5b) and the findings of previous studies (Lee et al., 2019; Min et al., 2013; Smith et al., 2008).

Within the discriminant validity domain, we explored associations between central measures of resilience and depression, anxiety, and maladaptive coping strategies. Both measures of resilience were negatively related to depression and anxiety, supporting our hypotheses (H6 and H7, respectively) based on previous studies (Smith et al., 2008; Yıldırım et al., 2020). Similarly, both measures of resilience were negatively related to both maladaptive coping strategies (i.e., self-blame and catastrophizing), supporting our hypotheses (H8a and H8b) and results of previous studies (Min et al., 2013; Lee et al., 2019).

Furthermore, we explored associations of both resilience measures with specific outcomes and their incremental validity. Both measures exhibited expected positive associations with all four QoL aspects (i.e., physical, psychological, social, and environmental). The results of regression analyses showed that both measures explained a significant share of variance in four QoL aspects above demographic variables and coping strategies, supporting our hypotheses (H9a, H9b, H9c, and H9d), which was based on previous studies (Keener et al., 2021; Tempski et al., 2015). As already pointed out in the results section, when we added an additional step in regression analysis to control for another measure of resilience, CYRM-12 still predicted all QoL outcomes, while BRS predicted only the psychological and environmental aspects of QoL.

The analyses of the nomological network of resilience revealed somewhat different results for CYRM-12 and BRS. While associations for both measures were generally in line with our hypotheses, they differed in their strength. Namely, in the case of convergent validity, BRS showed weaker associations with positive coping strategies than CYRM-12. In comparison, in the case of discriminant validity, there were somewhat stronger associations of BRS with anxiety and maladaptive coping strategies than of CYRM-12 with the same constructs. The most notable difference emerged when exploring incremental validity. After controlling for demographic characteristics, coping strategies, and another measure of resilience, BRS predicted only psychological and environmental aspects of QoL, while CYRM-12 still predicted all four. Therefore, we can conclude that associations with other constructs provide stronger support for the validity of CYRM-12 compared to BRS. Despite our best efforts, we have not found any previous studies examining both CYRM-12 and BRS simultaneously in relation to the constructs explored in this study. However, two reviews (Ballard et al., 2024; Windle et al., 2011) evaluated the psychometric and other characteristics of BRS and the longer version of CYRM, from which the CYRM-12 was derived (Liebenberg et al., 2013). Both reviews evaluated several resilience questionnaires against the predefined criteria regarding questionnaires’ development, assessment of psychometric structure, and validity evidence. In general, CYRM was one of the questionnaires that were awarded the most points in the review, while BRS was the least favorably evaluated measure by Ballard et al. (2024), while in the Windle et al. (2011) review, BRS received a better general evaluation than CYRM. More specifically, CYRM outperformed BRS regarding the criteria of theory formulation (Ballard et al., 2024) and content validity (Windle et al., 2011), while BRS outperformed CYRM in criteria regarding discriminant validity evidence (Ballard et al., 2024), internal consistency, construct validity evidence, reproducibility and interpretability (Windle et al., 2011). While the central measures differ in some aspects of their psychometric evaluation, the most important difference stems from the focus of both questionnaires; BRS was developed to evaluate the ability to recover or bounce back after experiencing stress, while the CYRM authors defined resilience as an interplay between resources and an individual (Windle et al., 2011). Therefore, we can conclude that while results support our hypotheses regarding the different types of validity for both Slovenian versions of the two questionnaires, some caution is advised when deciding on their use. This is particularly the case for authors interested in QoL outcomes, as CYRM was related to all four aspects when controlling for other variables and another resilience measure, while for BRS, this held true only for the psychological and environmental aspects of QoL.

### Implications

The implications of the present study highlight robust psychometric properties of the Slovenian versions of the CYRM-12 and BRS resilience measures among youth. Our findings support the unidimensional structure of both scales, along with their convergent, discriminant, and incremental validity, suggesting that these measures can effectively assess resilience in this demographic. Notably, the CYRM-12 demonstrated a stronger predictive value for quality of life outcomes compared to the BRS, indicating its potential as a more comprehensive measure for evaluating resilience in relation to these outcomes. The positive associations with adaptive coping strategies and negative correlations with depression and anxiety further underscore the importance of resilience in mental health among Slovenian youth. These results pave the way for future research on resilience, enabling cross-cultural comparisons and enhancing the resources available to practitioners for evidence-based interventions aimed at fostering resilience in young individuals.

### Limitations and future research

The present study has some limitations. First, as the collected data are solely based on self-report, the study is not immune to the well-known shortcomings of this approach, including social desirability, which can create false relationships or obscure relationships between variables (van de Mortel, 2008) and recall biases, which can lead to under- or overestimation of positive or negative past emotional experiences (Colombo et al., 2020). Second, as the study was conducted online, we could not control the external factors that may disrupt participants, such as noise, interruptions, or other distractions. Third, we recruited participants via an online panel, which could affect different aspects of data quality, such as attention, comprehension, honesty, and reliability (Peer et al., 2021). However, this problem was mitigated by employing attention checks in the form of directed questions (Maniaci and Rogge, 2014). Fourth, we employed a convenience sample of young individuals willing to participate in the study in exchange for a smaller reward. While the resulting sample is relatively diverse, it is only partially representative as, for example, demonstrated by a larger share of women (63.3%) than men (35.2%). This may hinder the generalizability of our findings. Lastly, even though our findings are consistent with those seen in other validations of the primary questionnaires, we did not specifically assess the measurement invariance of the translated and original versions.

Future research may replicate and extend our study by recruiting a representative sample of young individuals or investigating the psychometric characteristics of the two central questionnaires in general population samples. Furthermore, future studies may focus on empirically investigating the measurement equivalence of the Slovenian versions and the original versions of both scales. Beyond psychometric studies, future research can build on our study and use the translated measures to investigate the protective role of resilience in coping with different stressors, explore cross-cultural differences in youth resilience, and quantify the effectiveness of resilience-building interventions. It is worth noting that some of these ideas will be explored in the EU funded project “*Supporting mental health in young people: Integrated Methodology for clinical decisions and evidence-based interventions (SMILE)*,” which will deploy digital tools capable of enhancing the resilience of young individuals in seven countries including Slovenia.

## Conclusion

In conclusion, our findings show that the Slovenian versions of CYRM-12 (Liebenberg et al., 2013) and BRS (Smith et al., 2008) are appropriate for use in future research investigating youth resilience, with CYRM-12 demonstrating somewhat larger predictive value for quality of life outcomes. Nonetheless, both measures of resilience exhibited the proposed one-factor structure, positive associations with other measures of resilience and adaptive coping strategies, negative associations with depression, anxiety, and maladaptive coping strategies, and positively predicted different aspects of quality of life. Hence, our study represents an important contribution to the literature, paving the way for further studies of resilience in Slovenia, facilitating cross-cultural comparisons, broadening the array of tools available to practitioners, and allowing for evidence-based policies aimed at enhancing resilience among youth.

## Data Availability

The raw data supporting the conclusions of this article will be made available by the authors, without undue reservation.

## Footnotes

^1^ https://www.valicon.net/

^2^ https://1ka.arnes.si/
