# Supplementary material for: Validation of the Slovenian versions of Child and Youth Resilience Measure-12 and Brief Resilience Scale among youth
Source: Front Psychol. 2025 Feb 19;16:1467174. doi: 10.3389/fpsyg.2025.1467174 (PMC11879954; doi:10.3389/fpsyg.2025.1467174)
Supplement: Supplementary file 1 [file Table_1.DOCX]

Supplementary Material

Table S1

*Incremental validity of CYRM-12 in predicting quality of life, after additionally controlling for CD-RISC-10*

|  | QOL: Physical | | | | QOL: Psychological | | | | QOL: Social | | | | QOL: Environmental | | | |
| --- | --- | --- | --- | --- | --- | --- | --- | --- | --- | --- | --- | --- | --- | --- | --- | --- |
|  | S1 | S2 | S3 | S4 | S1 | S2 | S3 | S4 | S1 | S2 | S3 | S4 | S1 | S2 | S3 | S4 |
| Gender | -.25^***^ | -.18^***^ | -.16^**^ | -.20^***^ | -.15^**^ | -.07 | -.04 | -.08 | .07 | .13^*^ | .15^**^ | .10^*^ | -.11^*^ | -.05 | -.03 | -.08 |
| Age | -.04 | -.05 | -.02 | .01 | .00 | -.01 | .04 | .06 | .03 | .02 | .05 | .08 | -.08 | -.08 | -.04 | -.02 |
| Education | .08 | .07 | .04 | .01 | .06 | .05 | .01 | -.03 | .08 | .07 | .04 | -.01 | .05 | .04 | .00 | -.04 |
| Socioeconomic status | .15^**^ | .10^*^ | .10^*^ | .09^*^ | .16^**^ | .09^*^ | .08^*^ | .07 | .07 | .02 | .01 | .00 | .24^***^ | .21^***^ | .20^***^ | .20^***^ |
| Refocus on planning |  | .01 | -.03 | -.06 |  | .08 | .01 | -.02 |  | .04 | -.01 | -.04 |  | .12^*^ | .07 | .04 |
| Positive reappraisal |  | .27^***^ | .10 | .05 |  | .38^***^ | .12^*^ | .06 |  | .27^***^ | .10 | .03 |  | .14^*^ | -.05 | -.12 |
| Self-blame |  | -.10 | -.07 | -.05 |  | -.18^***^ | -.14^**^ | -.12^**^ |  | -.12^*^ | -.09 | -.06 |  | -.07 | -.05 | -.02 |
| Catastrophizing |  | -.23^***^ | -.15^**^ | -.12^*^ |  | -.25^***^ | -.13^**^ | -.10^*^ |  | -.20^***^ | -.12^*^ | -.08 |  | -.21^***^ | -.13^*^ | -.08 |
| Resilience (CD-RISC-10) |  |  | .34^***^ | .20^**^ |  |  | .51^***^ | .36^***^ |  |  | .33^***^ | .13 |  |  | .37^***^ | .18^**^ |
| Resilience (CYRM-12) |  |  |  | .33^***^ |  |  |  | .35^***^ |  |  |  | .47^***^ |  |  |  | .44^***^ |
| *R*^2^ | .093 | .269 | .328 | .392 | .053 | .382 | .514 | .588 | .020 | .189 | .244 | .372 | .080 | .197 | .266 | .382 |
| *F*^a^ | 8.24^***^ | 14.51^***^ | 17.06^***^ | 20.28^***^ | 4.47^**^ | 24.46^***^ | 37.06^***^ | 44.77^***^ | 1.59 | 9.20^***^ | 11.32^***^ | 18.59^***^ | 6.94^***^ | 9.69^***^ | 12.69^***^ | 19.42^***^ |
| *ΔR*^2^ | .093 | .175 | .059 | .065 | .053 | .330 | .132 | .073 | .020 | .169 | .056 | .127 | .080 | .117 | .069 | .116 |
| *ΔF*^b^ | 8.24^***^ | 18.93^***^ | 27.67^***^ | 33.45^***^ | 4.47^**^ | 42.18^***^ | 85.56^***^ | 55.96^***^ | 1.59 | 16.50^***^ | 23.14^***^ | 63.69^***^ | 6.94^***^ | 11.52^***^ | 29.67^***^ | 59.01^***^ |

*Notes. N* = 325 as we only included those who identified as male (0) or female (1) in these analyses. S1 = Step 1, S2 = Step 2, S3 = Step 3, S4 = Step 4. ^a^ Degrees of freedom were 4, 320 in Step 1; 8, 316 in Step 2; 9, 315 in Step 3; and 10, 314 in Step 4. ^b^ Degrees of freedom were 4, 320 in Step 1; 4, 316 in Step 2; 1, 315 in Step 3; and 1, 314 in Step 4.

Table S2

*Incremental validity of BRS in predicting quality of life, after additionally controlling for CD-RISC-10*

|  | QOL: Physical | | | | QOL: Psychological | | | | QOL: Social | | | | QOL: Environmental | | | |
| --- | --- | --- | --- | --- | --- | --- | --- | --- | --- | --- | --- | --- | --- | --- | --- | --- |
|  | S1 | S2 | S3 | S4 | S1 | S2 | S3 | S4 | S1 | S2 | S3 | S4 | S1 | S2 | S3 | S4 |
| Gender | -.25^***^ | -.18^***^ | -.16^**^ | -.15^**^ | -.15^**^ | -.07 | -.04 | -.02 | .07 | .13^*^ | .15^**^ | .16^**^ | -.11^*^ | -.05 | -.03 | -.01 |
| Age | -.04 | -.05 | -.02 | -.01 | .00 | -.01 | .04 | .04 | .03 | .02 | .05 | .05 | -.08 | -.08 | -.04 | -.04 |
| Education | .08 | .07 | .04 | .04 | .06 | .05 | .01 | .02 | .08 | .07 | .04 | .04 | .05 | .04 | .00 | .01 |
| Socioeconomic status | .15^**^ | .10^*^ | .10^*^ | .09 | .16^**^ | .09^*^ | .08^*^ | .06 | .07 | .02 | .01 | .01 | .24^***^ | .21^***^ | .20^***^ | .19^***^ |
| Refocus on planning |  | .01 | -.03 | -.03 |  | .08 | .01 | .01 |  | .04 | -.01 | -.01 |  | .12^*^ | .07 | .07 |
| Positive reappraisal |  | .27^***^ | .10 | .11 |  | .38^***^ | .12^*^ | .13^*^ |  | .27^***^ | .10 | .10 |  | .14^*^ | -.05 | -.03 |
| Self-blame |  | -.10 | -.07 | -.07 |  | -.18^***^ | -.14^**^ | -.14^***^ |  | -.12^*^ | -.09 | -.09 |  | -.07 | -.05 | -.05 |
| Catastrophizing |  | -.23^***^ | -.15^**^ | -.13^*^ |  | -.25^***^ | -.13^**^ | -.10^*^ |  | -.20^***^ | -.12^*^ | -.12 |  | -.21^***^ | -.13^*^ | -.10 |
| Resilience (CD-RISC-10) |  |  | .34^***^ | .29^***^ |  |  | .51^***^ | .42^***^ |  |  | .33^***^ | .31^***^ |  |  | .37^***^ | .29^***^ |
| Resilience (BRS) |  |  |  | .09 |  |  |  | .15^**^ |  |  |  | .04 |  |  |  | .15^*^ |
| *R*^2^ | .093 | .269 | .328 | .333 | .053 | .382 | .514 | .527 | .020 | .189 | .244 | .245 | .080 | .197 | .266 | .278 |
| *F*^a^ | 8.24^***^ | 14.51^***^ | 17.06^***^ | 15.64^***^ | 4.47^**^ | 24.46^***^ | 37.06^***^ | 35.05^***^ | 1.59 | 9.20^***^ | 11.32^***^ | 10.21^***^ | 6.94^***^ | 9.69^***^ | 12.69^***^ | 12.08^***^ |
| *ΔR*^2^ | .093 | .175 | .059 | .005 | .053 | .330 | .132 | .013 | .020 | .169 | .056 | .001 | .080 | .117 | .069 | .012 |
| *ΔF*^b^ | 8.24^***^ | 18.93^***^ | 27.67^***^ | 2.28 | 4.47^**^ | 42.18^***^ | 85.56^***^ | 8.73^**^ | 1.59 | 16.50^***^ | 23.14^***^ | 0.36 | 6.94^***^ | 11.52^***^ | 29.67^***^ | 5.13^*^ |

*Notes. N* = 325 as we only included those who identified as male (0) or female (1) in these analyses. S1 = Step 1, S2 = Step 2, S3 = Step 3, S4 = Step 4. ^a^ Degrees of freedom were 4, 320 in Step 1; 8, 316 in Step 2; 9, 315 in Step 3; and 10, 314 in Step 4. ^b^ Degrees of freedom were 4, 320 in Step 1; 4, 316 in Step 2; 1, 315 in Step 3; and 1, 314 in Step 4.
